# Ring and Gellhorn pessaries used in patients with pelvic organ prolapse: a retrospective study of 8 years

**DOI:** 10.1007/s00404-018-4844-z

**Published:** 2018-07-05

**Authors:** Junfang Yang, Jinsong Han, Fuli Zhu, Yu Wang

**Affiliations:** 1 Department of Obstetrics and Gynecology, Peking University Third Hospital, Garden North Road No. 49, Haidian District, Beijing, 100191 China; 2 Department of Obstetrics and Gynecology, China Academy of Chinese Medical Sciences Xiyuan Hospital, Beijing, China; 3 Department of Obstetrics and Gynecology, Peking University First Hospital, Beijing, China

**Keywords:** Pelvic organ prolapse, Pessary fitting, Satisfaction, Complication

## Abstract

**Aim:**

The aim of this study was to identify factors associated with pessary fitting, continued use of pessary and satisfaction of patients with pelvic organ prolapse.

**Methods:**

A retrospective study was conducted in patients who received an initial pessary fitting. The clinical characteristics of these patients were recorded. The Pelvic Floor Disability Index PFDI-20 and PFIQ-7 were used to assess pelvic floor dysfunction and quality of life. Complications, satisfaction degree, and reasons for abandonment were recorded during the follow-up. *T* test and Chi square test in SPSS version 20 were used to analyze the data.

**Results:**

Three hundred women with symptomatic prolapse were selected for pessary fitting, whose average age was 67.8 ± 10.7 years. For two hundred and forty-nine (83%) women, the fitting was successful, of whom 162 used ring pessaries and 87 used Gellhorn pessaries. Forty-seven patients abandoned using a pessary at the end of our study. Most clinical characteristics were not significantly different between the successful and unsuccessful fitting groups (*P *> 0.05). The average score of CRADI-8 was lower in successful fitting group (11.9 ± 15.9) than that in unsuccessful fitting group (18.8 ± 19.9) (*P *< 0.05). 162 patients with successful pessary fitting completed the satisfaction survey, 79% of whom were satisfied or very satisfied. Erosions (24.4%) were the most common complication. Difficulty in inserting or removing (30.4%) and erosions (22.8%) were the main factors, which affected the satisfaction degree.

**Conclusions:**

Patients with obvious symptoms of posterior pelvic prolapse are more likely to fit failure. Difficulty in inserting or removing and erosions are the main factors, which lead to the discontinuation of pessary use and decrease in the satisfaction degree.

**Electronic supplementary material:**

The online version of this article (10.1007/s00404-018-4844-z) contains supplementary material, which is available to authorized users.

## Introduction

Pelvic organ prolapse is a common disease worldwide. Its prevalence rate is reported to be 41% in postmenopausal women in the US [1]. The prevalence of typical prolapse symptoms is reported to be 12% in women in the US [2]. It is projected that the number of women with pelvic organ prolapse will increase to 46% between 2010 and 2050 [3]. There are conservative and surgical treatments for symptomatic prolapse. Pessary is an important conservative treatment option that is recommended as the first-line treatment by 77% of the members of the American urogynecologic association [4]. Pessaries can be categorized into two types, supporting type and space-occupying type. Ring pessaries are the supporting type. They are commonly recommended for stage I or II prolapse. However, ring pessaries with support can be successfully used in patients with stage III or IV prolapse [5]. Gellhorn pessaries have the functions of supporting and space-occupying. They are often used to treat advanced prolapse.

In our study, we mainly used the ring and Gellhorn pessaries, since they were easy to fit and follow-up for Chinese patients. The purpose of this study was to compare the differences in patients’ clinical characteristics between the successful and unsuccessful pessary fitting groups. In addition, we documented the pessary failure, complications and patients’ satisfaction during the follow-up.

## Materials and methods

### Study population

This was a retrospective clinical study of patients who presented for an initial pessary fitting at the outpatient gynecology clinic of Peking University Third Hospital from January 2008 to July 2016. All the patients with symptomatic prolapse (defined as the feeling of bulging or protrusion from the vagina) were selected to undergo a pessary trial after discussion of all therapeutic options (observation, pessary and surgery). Patients who met the following criteria underwent a pessary trial. (1) Cervical cytological examination was normal. (2) There was no inflammation in the genital organs. (3) Patient was not allergic to silicone. During the 8 years, the indication and counseling algorithms were not changed.

### Pessary type

The silicone pessaries used in our department, were all produced by American COOPER Company. The main types of pessary were the ring with support and Gellhorn. The diameters of the ring pessary included 57, 64, 70, 76, and 83 mm. The diameters of the Gellhorn pessary included 44, 51, 57, 64, 70, and 76 mm.

### Pessary fitting procedure

Pessary trials were performed by a trained physician. The first choice was a ring pessary with support. If a ring pessary with support could not be fitted, a Gellhorn pessary was tried. A pessary was considered to be the correct size when the physician could place a single finger between the pessary and the vaginal wall. And the prolapse was reduced to above the hymen. Patients should feel comfortable without dysuria. The pessary should be retained during a Valsalva maneuver, coughing and walking. Women and their families were taught to insert and take out a pessary. Then, the fitted pessaries were taken home by patients. The pessaries would be placed in the vagina less than half an hour every day. After a week, an appointment was scheduled to evaluate the fit. Patients whose pessary fell out or who experienced discomfort within the first week were assisted to refit with a different type or size of pessary. The result was reviewed again after another week. Successful pessary fitting was defined as retaining the pessary for 1 week without any discomfort [6]. The unsuccessful fitting was usually due to failure to find appropriate size, discomfort, difficulty in inserting and taking out, or other factors that caused patients to abandon a pessary.

### Data collection

The data was collected by paper-based records. PFDI-20 and PFIQ-7 were evaluated before the pessary trial. Patients who had been fitted successfully were asked to come back to our outpatient department every 6 months. Follow-up was conducted through telephone interview at the end of our study. Complications, satisfaction degree, and reasons for abandonment were recorded during the follow-up. Pessary failure was defined as discontinuation of pessary use at any time after successful fitting.

The Pelvic Floor Distress Inventory-20 (PFDI-20) and the Pelvic Floor Impact Questionnaire-7 (PFIQ-7) [7] were used to assess pelvic floor dysfunction and quality of life, with higher scores indicating greater impact. PFDI-20 includes three subscales: the Pelvic Organ Prolapse Distress Inventory-6 (POPDI-6), for prolapse symptoms; the Colorectal-Anal Distress Inventory-8 (CRADI-8), for colorectal/anal symptoms; and the Urinary Distress Inventory-6 (UDI-6), for urinary symptoms. The PFIQ-7 also includes three subscales: the Urinary Impact Questionnaire-7 (UIQ-7), the Colorectal Impact Questionnaire-7 (CRAIQ-7), and the Pelvic Organ Prolapse Impact Questionnaire-7 (POPIQ-7). Satisfaction degree was calculated by the addition of each subjective degree and was expressed as very unsatisfied (1 point), unsatisfied (2 point), average (3 points), satisfied (4 points), and very satisfied (5 points).

### Analysis

All the measured data were tested in normality. The normal distribution was analyzed by independent sample *T* test. Descriptive statistics were used to report quantitative values as means ± standard deviations (SD). Chi square test was used for enumeration data. A result was considered statistically significant when *P *< 0.05. All analyses were performed using SPSS version 20.

## Results

**Fig. 1:**
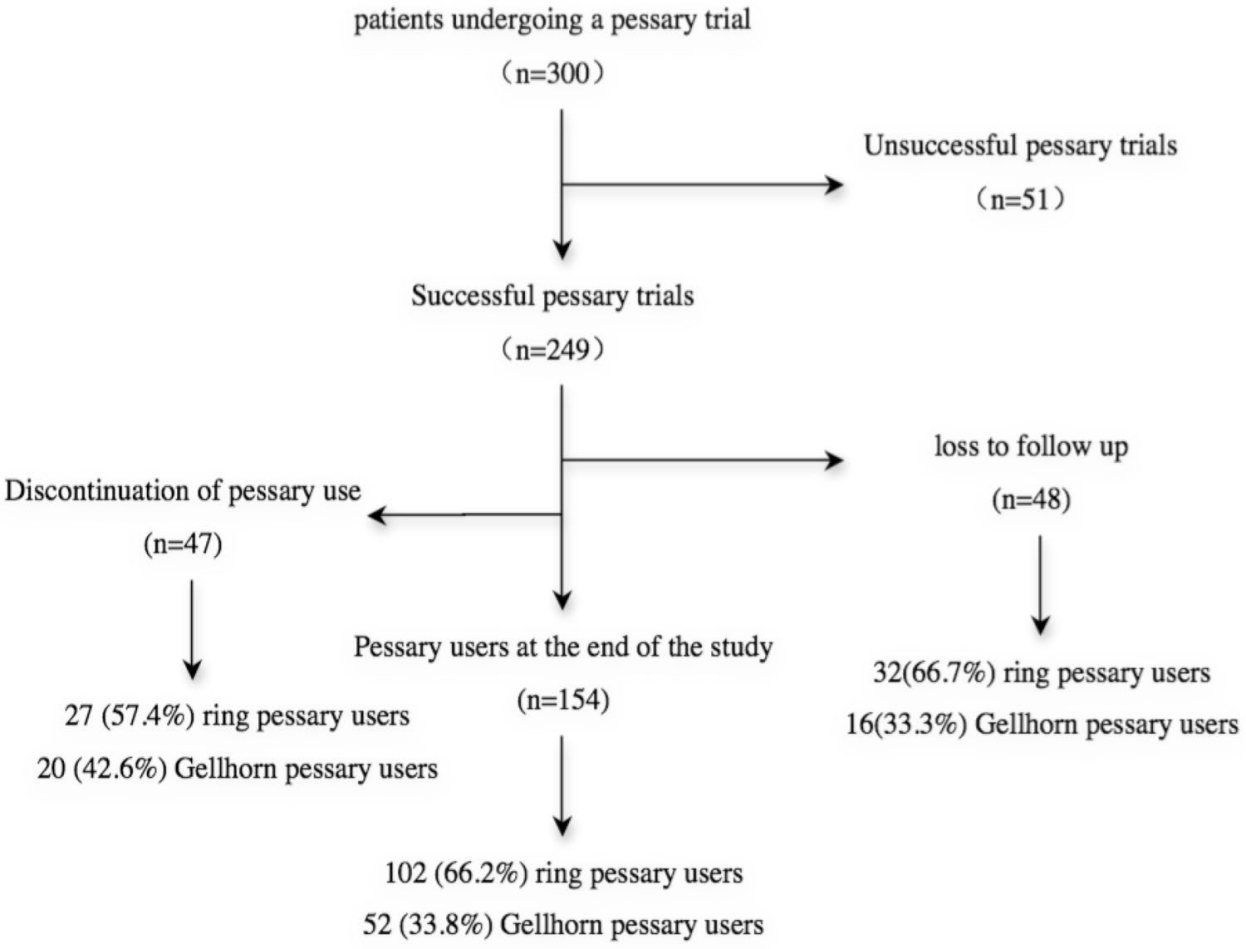
Flow diagram showing the outcomes of the pessary fittings and subsequent use

From January 2008 to July 2016, 300 women with symptomatic prolapse were selected to try a pessary, whose average age was 67.8 ± 10.7 years. 4% (12/300) of them were premenopausal, 3.0% (9/300) underwent hysterectomy for other disease, 1.3% (4/300) had a recurrence after pelvic floor reconstructive surgery (three women with mesh repair of anterior vaginal wall and one woman with traditional vaginal repair). Except for 10 women with stage II, the POP-Q staging conditions of most women were stage III or IV. Figure 1 shows the outcomes of the pessary fittings and subsequent use. Pessary fitting was successful in 83% (249/300). The abandon rates of ring pessary and Gellhorn pessary were 20.9% (27/129) and 27.8% (20/72).

**Fig. 2:**
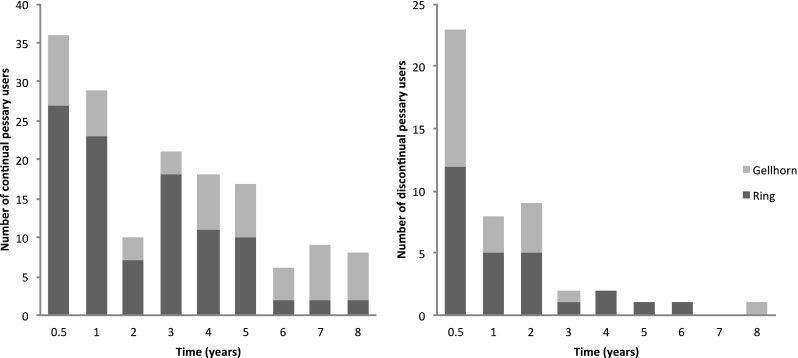
Column diagram showing the distribution of continual and discontinual users with successful pessary trials during the 8 years. (The left diagram shows the distribution of continual pessary users and the right diagram shows the distribution of discontinual pessary users)

The follow-up results of patients with successful pessary trials were shown in Fig. 2. A total of 47 patients gave up the use of pessary. Twenty (42.6%) of them gave up in the first 6 months. In our results, the rates of the continuous use of all types of pessaries were 86.1% (142/165) after 1 year, 77.2% (105/136) after 2 years, and 49.4% (43/87) after 5 years.

**Fig. 3:**
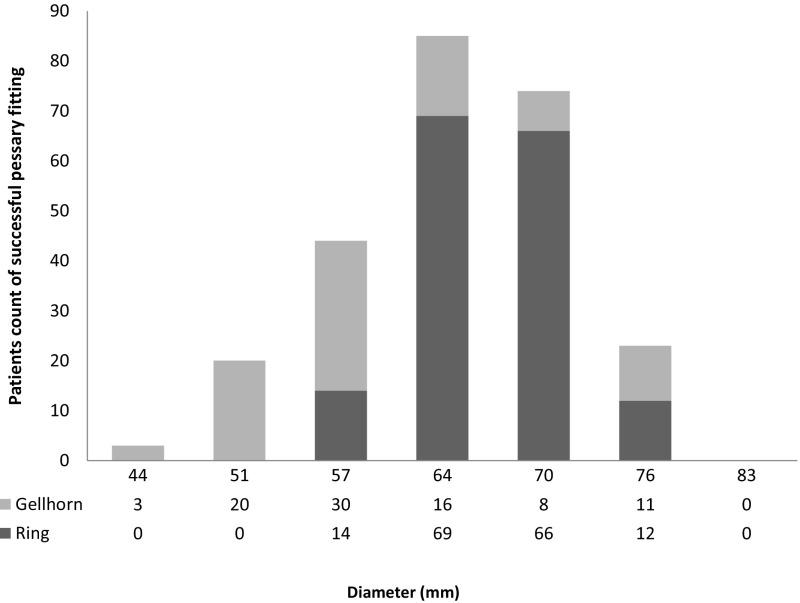
The number of two types of pessary used in patients of successful fitting

In our study, we found that the Gellhorn pessaries of 51 mm (22.7%), 57 mm (34.1%), and 64 mm (18.2%) in size were the most commonly used. The most commonly used ring pessaries are 64 mm (42.9%) and 70 mm (41.0%) (Fig. 3).

We compared the differences in patients’ clinical characteristics between the successful and unsuccessful pessary fitting groups (Table 1). All of these clinical characteristics were similar between the two groups with no statistical difference (*P *> 0.05) except for CRADI-8. The average score of CRADI-8 of the successful pessary fitting group was significantly lower than that of the unsuccessful pessary fitting group (*P *< 0.05).

**Table 1:** Comparison of characteristics between successful and unsuccessful pessary fitting groups

	Successful pessary fitting (*n* = 249)	Unsuccessful pessary fitting (*n* = 51)	*P*
Age (years)	68.2 ± 10.1	65.6 ± 13.1	0.18
BMI (kg/m^2^)	23.9 ± 2.8	24.6 ± 2.7	0.12
Age of menopause (years)	49.9 ± 3.7	50.2 ± 4.3	0.70
Gestation	3.5 ± 1.5	3.2 ± 1.5	0.32
Parity	2.5 ± 1.3	2.1 ± 1.4	0.05
Age of onset (years)	63.8 ± 12.4	60.9 ± 14.4	0.15
History of hysterectomy	7 (2.8)	2 (3.9)	0.70
Hypertension	144 (57.8)	22 (43.1)	0.05
Diabetes	53 (21.3)	6 (11.8)	0.12
Heart disease	77 (30.9)	13 (25.6)	0.43
PFDI-20	74.7 ± 47.5	86.5 ± 50.1	0.19
POPDI-6	32.9 ± 19.1	36.7 ± 19.3	0.28
UDI-6	30.2 ± 24.6	30.9 ± 18.7	0.87
CRADI-8	11.9 ± 15.9	18.8 ± 19.9	0.03
PFIQ-7	51.3 ± 50.2	44.9 ± 38.5	0.48
POPIQ	29.5 ± 27.7	26.1 ± 25.1	0.51
UIQ	16.7 ± 24.9	14.0 ± 17.0	0.55
CRAIQ	5.0 ± 14.1	4.8 ± 9.9	0.93

Data are mean ± standard deviation or *n* (%)

*BMI* Body Mass Index

For patients with successful pessary fitting, complications were erosions (24.4%), abnormal vaginal bleeding (9.5%), urinary incontinence (3.0%), vaginitis (2.5%), voiding difficulty (2.0%), defecation difficulty (1.5%), fecal incontinence (0.5%), allergy (0.5%), and lower back pain (0.5%). All the complications were mild, and could be relieved by drug treatment or changing pessaries. None of the patients had severe complications. One hundred and sixty-two patients with successful pessary fitting completed the satisfaction survey. Eleven cases (6.8%) were rated 1 point, 8 cases (4.9%) were rated 2 points, 15 cases (9.3%) were rated 3 points, and 58 cases (35.8%) were rated 4 points. Seventy cases (43.2%) were rated 5 points. We also documented the symptoms of patients during the follow-up, including the factors that affected satisfaction and caused the patients to abandon their pessaries (Table 2). During our follow-up, a fraction of the patients were reluctant to answer questions, therefore, we were not able to find out the reasons for them to abandon their pessaries.

**Table 2:** Symptoms during the follow-up that decreased patients’ satisfaction degree and caused patients to remove a pessary

Symptoms	Affecting factors of satisfaction (*n* = 92)	Reasons of removing pessary (*n* = 47)
Erosions	21	6
Abnormal vaginal bleeding	3	
Urinary incontinence	5	
Defecation difficulty	3	1
Voiding difficulty	3	2
Vaginitis	2	2
Fecal incontinence allergy	1	1
Difficulty in inserting or removing	28	9
Falling out	14	4
Discomfort	9	3
Fear of foreign-body reaction	3	
Symptoms not improved		4
Unknown reason		15

## Discussion

In our results, the rates of continuous use of pessary were 86.1% after 1 year, 77.2% after 2 years, and 49.4% after 5 years. A 12-year study of Sophie et al. showed that for women aged 65–74 years and women aged 75 years and older, the cumulative probabilities of continuous pessary use were 87.5% and 80.8% after 1 year, 80.6 and 70.9% after 2 years, and 62.1 versus 37.8% after 5 years [8]. We found 18.9% of the patients discontinued the use of a pessary. 42.6% (20/47) of them gave up in the first 6 months. In Lone F’s study, most failures (73.8%) occurred within 4 weeks of pessary insertion, and they thought complications might be the reason [9]. A retrospective chart review showed that the most common reasons for discontinuation of pessary use were discomfort (35%), falling out (17%), erosions (14%), desires surgery instead (11%), bleeding (7%), symptoms not improved with pessary (6%), and incontinence (6%) [10]. Bai et al. investigated 104 patients fitted with pessaries, of whom 19.1% removed their pessaries. Most patients (80.0%) were unable to continue use due to the repeated expulsion of the pessary and uncomfortable fitting [11]. We found difficulty in inserting or removing (19.1%). Erosions (12.8%) were the main reason of failing. A questionnaire survey of 947 gynecologists reported that the ring pessary was the most commonly used one by clinicians and the easiest to insert and remove. The Gellhorn pessary was the most effective in pelvic organ prolapse (POP), but the most difficult to remove [12]. Therefore, follow-up and guidance are critical in the first 6 months. For most patients who were elderly with poor self-care ability, it is very important for the physician to teach the patient how to insert and remove the pessary in our clinical work.

Researches about predictors of successful pessary fitting can not reach an agreement due to the potential selection bias. Some studies indicated that predominant prolapse of the anterior wall and longer vaginal length might help hold the pessary in the vagina, which might influence successful pessary fitting [13–15]. In addition, a short vagina and wide vaginal hiatus [16–18], posterior wall prolapse [19], previous prolapse repair, and hysterectomy [18] might be associated with unsuccessful fitting. Cheung et al. indicated that levator ani muscle (LAM) avulsion increased the risk of expulsion of vaginal pessary [15]. A cross-sectional study showed factors associated with unsuccessful pessary fitting were age, body mass index, and having underactive pelvic floor muscles [6]. Our results showed that age, body mass index, history of hysterectomy, and questionnaire of PFIQ-7 were not significantly different between the successful and unsuccessful fitting groups. The average score of CRADI-8 was lower in successful fitting group than that in unsuccessful fitting group (*P *< 0.05). However, the average score of CRAIQ was not significantly different between the two groups (*P *> 0.05). CRADI-8 was related to the severity of symptoms of posterior pelvic prolapse. CRAIQ was related to the effects of posterior pelvic prolapse on the quality of life. The results indicated that patients with obvious symptoms of posterior pelvic prolapse were more likely to experience fitting failure, although these symptoms did not result in a significant difference in the quality of life between the two groups.

Pessary fitting was successful in 83% in our study, indicating that pessary treatment could be accepted by most women with symptomatic prolapse. In outpatient department, we explained the advantages and disadvantages of different treatment options, which included observation, pessary and surgery, and let the patients chose by themselves without clinicians’ recommendations. Therefore, all the patients who chose pessary had good psychological acceptance, which might be the reason for the higher rate of successful pessary fitting in our study.

Two types of pessaries were included in our study. Because the ring pessary was easier to insert and take out, the first choice was a ring pessary with support, and a Gellhorn pessary was tried following a failed fitting of a ring pessary with support. It is expected that more patients used ring pessaries than those using Gellhorn pessaries. In addition, the abandon rate of ring pessaries was lower than that of Gellhorn pessaries, which indicated that the tolerance of patients to ring pessary was better than that of Gellhorn pessary. 83.9% of the patients with a ring pessary were using size 64 or 70 mm in diameters. 75% of the patients with a Gellhorn pessary were treated with 51, 57 and 64 mm diameters. According to the results, we recommended clinicians firstly chose the intermediate diameter (ring 64 mm and Gellhorn 57 mm) in a pessary trial of Chinese women.

The symptoms induced using a pessary can be classified into erosion, infection, inflammation, neoplasia and occlusive symptoms [20]. An integrative review indicated that the most common complications reported were vaginal discharge/vaginitis, erosion, and bleeding. Complications were related to pessary shape and material, and duration in situ [21]. Sang et al. reported that 76 (73.1%) patients had complications such as bleeding, erosion, or foul odor [11]. Erosions and abnormal vaginal bleeding were the most common complications in our patients, which was consistent with the results reported in previous literature. 79% of the women who were successfully fitted expressed that they were satisfied or very satisfied with a pessary. 11.7% of the patients were unsatisfied or very unsatisfied. In some other studies, the satisfaction rates of pessary use were 70.2–92% [11, 22]. Although the complications were common, there were no severe complications. Most patients were still satisfied with the pessary. 30.4% (28/92) of the women with lower satisfaction complained about the difficulty in inserting or removing a pessary. 22.8% (21/92) of them thought erosions affected the quality of life. Thus, clinicians should focus on reducing the difficulty of pessary use in addition to treating the complications.

Our results are limited to the ring and Gellhorn pessary. The use of other types of pessaries has not been investigated. In our study, the first choice was a ring pessary with support, and a Gellhorn pessary was tried as a second choice. Therefore, this may have influenced the choice of pessary types and the outcome of our study.

## Conclusions

There are no significant differences in age, body mass index, history of hysterectomy, and stage of pelvic organ prolapse between the successful and unsuccessful pessary fitting groups in our study. Patients with obvious symptoms of posterior pelvic prolapse are more likely to experience unsuccessful pessary fitting. Complications caused by pessary use are common, but severe complications are rare. The follow-up and assistance in the first 6 months are critical for successful pessary use. The clinicians should focus on supporting patients to overcome the difficulties in pessary insertion and removal.

## Electronic supplementary material

Below is the link to the electronic supplementary material.

Supplementary material 1 (PPTX 108 kb)

## References

[1] Hendrix SL, Clark A, Nygaard I, Aragaki A, Barnabei V, McTiernan A (2002). Pelvic organ prolapse in the Women’s Health Initiative: gravity and gravidity. Am J Obstet Gynecol.

[2] Nygaard I, Barber MD, Burgio KL, Kenton K, Meikle S (2008). Prevalence of symptomatic pelvic floor disorders in US women. JAMA.

[3] Wu JM, Hundley AF, Fulton RG, Myers ER (2009). Forecasting the prevalence of pelvic floor disorders in U.S. Women: 2010 to 2050. Obstet Gynecol.

[4] Cundiff GW, Weidner AC, Visco AG, Bump RC, Addison WA (2000). A survey of pessary use by members of the American urogynecologic society. Obstet Gynecol.

[5] Ding J, Chen C, Song XC, Zhang L, Deng M, Zhu L (2016). Changes in prolapse and urinary symptoms after successful fitting of a ring pessary with support in women with advanced pelvic organ prolapse: a prospective study. Urology.

[6] Panman CM, Wiegersma M, Kollen BJ, Burger H, Berger MY, Dekker JH (2017). Predictors of unsuccessful pessary fitting in women with prolapse: a cross-sectional study in general practice. Int Urogynecol J.

[7] Barber MD, Walters MD, Bump RC (2005). Short forms of two condition-specific quality-of-life questionnaires for women with pelvic floor disorders (PFDI-20 and PFIQ-7). Am J Obstet Gynecol.

[8] Ramsay S, TulM Tannenbaum C (2016). Natural history of pessary use in women aged 65–74 versus 75 years and older with pelvic organ prolapse: a 12-year study. Int Urogynecol J.

[9] Lone F, Thakar R, Sultan AH, Karamalis G (2011). A 5-year prospective study of vaginal pessary use for pelvic organ prolapse. Int J Gynaecol Obstet.

[10] Wolff B, Oho AUID-, Williams K, Winkler A, Lind L, Shalom D (2017). Pessary types and discontinuation rates in patients with advanced pelvic organ prolapse. Int Urogynecol J.

[11] Bai SW, Yoon BS, Kwon JY, Shin JS, Kim SK, Park KH (2005). Survey of the characteristics and satisfaction degree of the patients using a pessary. Int Urogynecol J Pelvic Floor Dysfunct.

[12] Pott-Grinstein E, Newcomer JR (2001). Gynecologists’ patterns of prescribing pessaries. J Reprod Med.

[13] Deng M, Ding J, Ai F, Zhu L (2017). Clinical use of ring with support pessary for advanced pelvic organ prolapse and predictors of its short-term successful use. Menopause.

[14] Lekskulchai O, Wanichsetakul P (2015). Factors affecting successfulness of vaginal pessary use for the treatment of pelvic organ prolapse. J Med Assoc Thail.

[15] Cheung RYK, Lee JHS, Lee LL, Chung TKH, Chan SSC (2017). Levatorani muscle avulsion is a risk factor for expulsion within 1 year of vaginal pessary placed for pelvic organ prolapse. Ultrasound Obstet Gynecol.

[16] Clemons JL, Aguilar VC, Tillinghast TA, Jackson ND, Myers DL (2004). Risk factors associated with an unsuccessful pessary fitting trial in women with pelvic organ prolapse. Am J Obstet Gynecol.

[17] Manchana T (2011). Ring pessary for all pelvic organ prolapse. Arch Gynecol Obstet.

[18] Markle D, Skoczylas L, Goldsmith C, Noblett K (2011). Patient characteristics associated with a successful pessary fitting. Female Pelvic Med Reconstr Surg.

[19] Yamada T, Matsubara S (2011). Rectocoele, but not cystocele, may predict unsuccessful pessary fitting. J Obstet Gynaecol.

[20] Roberge RJ, Keller C, Garfinkel M (2001). Vaginal pessary-induced mechanical bowel obstruction. J Emerg Med.

[21] Abdulaziz M, Stothers L, Lazare D, Macnab A (2015). An integrative review and severity classification of complications related to pessary use in the treatment of female pelvic organ prolapse. Can Urol Assoc J.

[22] Clemons JL, Aguilar VC, Tillinghast TA, Jackson ND, Myers DL (2004). Patient satisfaction and changes in prolapse and urinary symptoms in women who were fitted successfully with a pessary for pelvic organ prolapse. Am J Obstet Gynecol.

