# Supplementary material for: Ring and Gellhorn pessaries used in patients with pelvic organ prolapse: a retrospective study of 8 years
Source: Arch Gynecol Obstet. 2018 Jul 5;298(3):623–9. doi: 10.1007/s00404-018-4844-z (PMC6096563; doi:10.1007/s00404-018-4844-z)
Supplement: Supplementary file 1 — Supplementary material 1 (PPTX 108 kb) [file 404_2018_4844_MOESM1_ESM.pptx]

## Slide 1
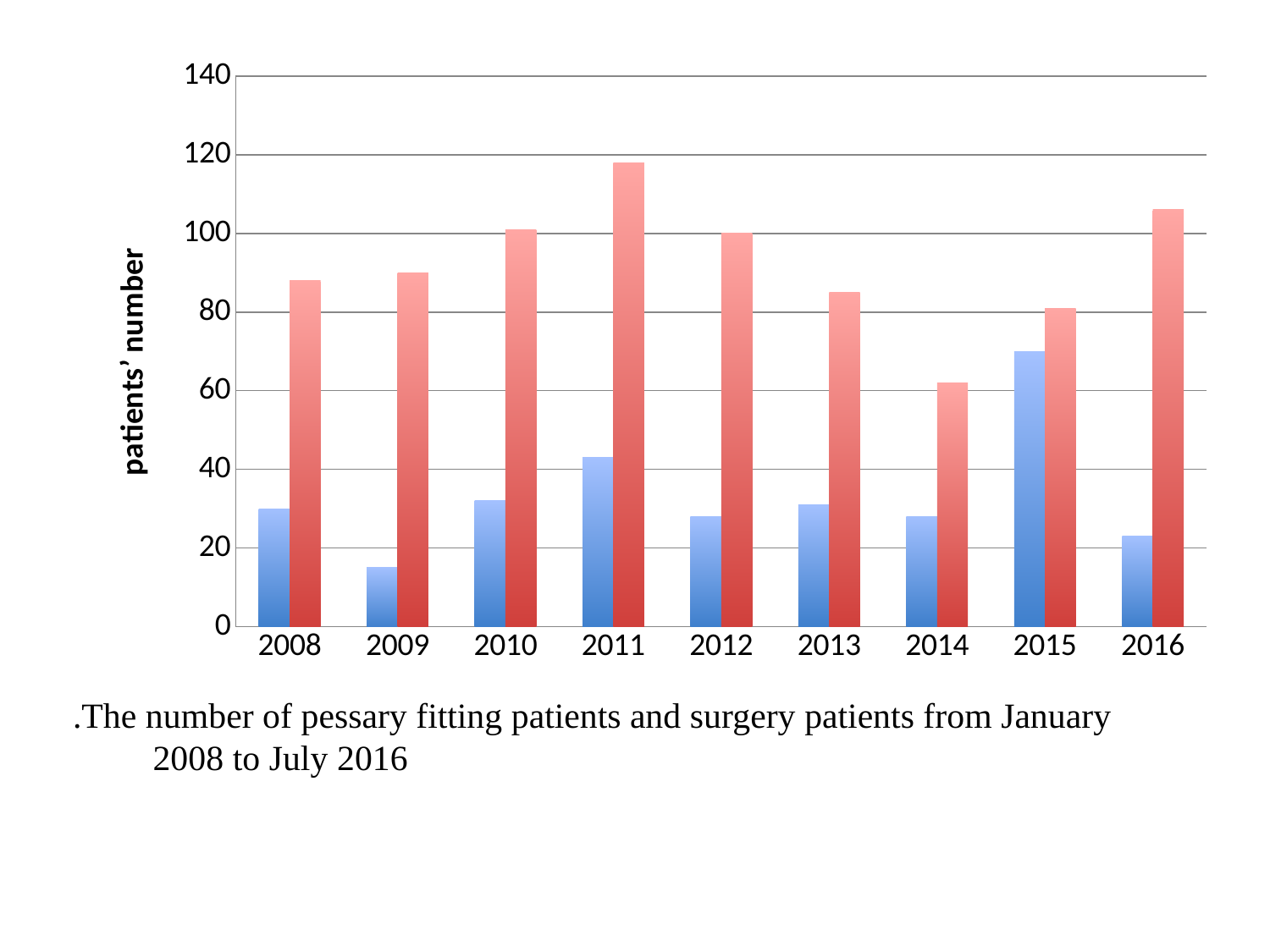

### Chart
| Category | pessary | surgery |
|---|---|---|
| 2008 | 30.0 | 88.0 |
| 2009 | 15.0 | 90.0 |
| 2010 | 32.0 | 101.0 |
| 2011 | 43.0 | 118.0 |
| 2012 | 28.0 | 100.0 |
| 2013 | 31.0 | 85.0 |
| 2014 | 28.0 | 62.0 |
| 2015 | 70.0 | 81.0 |
| 2016 | 23.0 | 106.0 |# .The number of pessary fitting patients and surgery patients from January 2008 to July 2016
